# Screening for Cognitive Impairment and Dementia Among Older People in Custody: A Systematic Review

**DOI:** 10.1177/14713012251356587

**Published:** 2025-06-26

**Authors:** Michael Chike Iloabachie, Bryce E. Stoliker, Lisa M. Jewell, Arlene Kent-Wilkinson

**Affiliations:** 1 School of Public Health, 7235 University of Saskatchewan, Saskatoon, SK, Canada; 2 Centre for Forensic Behavioural Science and Justice Studies, 7235 University of Saskatchewan, Saskatoon, SK, Canada; 3 College of Nursing, 7235 University of Saskatchewan, Saskatoon, SK, Canada

**Keywords:** dementia, cognitive assessment, screening tools, older people in custody, prison

## Abstract

Despite the growing number of older and aging people in custody across many countries, and corresponding vulnerability for neurocognitive issues, there has been limited research into the efficacy of cognitive assessment and dementia screening tools in correctional settings. The purpose of this study was to synthesize recent empirical research investigating dementia (and cognitive impairment) in older people in custody using screening tools. A systematic review methodology was adopted, with reporting guided by the PRISMA framework. This included a systematic search of four databases (PsycINFO, MedLine, PubMed, and Academic Search Complete) and handsearching of reference lists of eligible studies. A total of 9 peer-reviewed publications were included. All studies were from high income countries, including the United States (*n* = 2), Germany (*n* = 2), Canada (*n* = 1), England and Wales (*n* = 2), Australia (*n* = 1), and France (*n* = 1). These studies were synthesized according to the following themes: (1) use of screening tools with modification for the correctional setting; (2) use of screening tools without modification for the correctional setting; and, (3) innovative advances in cognitive screening tools and practices for correctional settings. This review identified several cognitive assessment and dementia screening tools that have been used in custodial settings. These tools can help in the early identification of dementia (or cognitive impairment) among older people in custody and, thus, inform the initiation of supports and strategies to manage those at risk. More research is needed to evaluate the performance of these tools compared with a diagnostic assessment.

## Introduction

Older and aging individuals^1^ represent a growing demographic in correctional institutions, which is a trend that has been well documented in several high-income countries (Baidawi et al., 2011; Beaudette & Stewart, 2014; Brooke et al., 2020; Forsyth et al., 2020, 2025; Gaston, 2018; Maschi et al., 2012; McKendy et al., 2019; Stoliker et al., 2022; Treacy et al., 2024). This growth has generally been attributed to overall trends in population aging and increased life expectancy, higher levels of crimes and arrests later in life, strict sentencing legislation and the imposition of longer sentences, and convictions for historical offences (Widra, 2023). Irrespective of the factors that have contributed to this demographic shift, correctional systems are faced with the challenge of addressing the complex health and social needs of older individuals within settings that are not designed for an aging population. One particular concern is the number of older people in custody at risk of, or living with, cognitive impairment and dementia and the suitability of prison environments for individuals with these health issues (Brooke et al., 2020; du Toit et al., 2019; Gaston, 2018; Maschi et al., 2012; Peacock et al., 2019; Treacy et al., 2024). The prevalence of cognitive impairment and dementia in older prison populations remains largely unknown (Brooke et al., 2020); however, estimates from international studies range anywhere from 1% to 70% (Ahalt et al., 2018; Combalbert et al., 2018; Forsyth et al., 2020, 2025; Perez et al., 2021; Stoliker et al., 2022; Verhülsdonk et al., 2021, 2023), suggesting a high level of variability potentially due to differences in study methodology (including the use of varying cognitive screening tools) and/or prison and population characteristics.

Despite the increased need for specialized care and resources in prisons to support older individuals with cognitive impairment and dementia, research on this topic has been limited (Forsyth et al., 2025). However, studies have emphasized the importance of early detection (and diagnosis) of cognitive impairment and dementia to initiate strategies to disrupt the progression of disease, as well as implementing care pathways to effectively manage and treat individuals presenting with these neurocognitive issues in custody and beyond (Forsyth et al., 2020; Gaston, 2018; MacRae et al., 2024; Stoliker et al., 2022; Treacy et al., 2024). Indeed, it has been recognized that cognitive screening tools should be incorporated as part of standard correctional healthcare policy and practice (Brooke et al., 2020; Peacock et al., 2019). Considering that early detection (and diagnosis) is a critical first step in establishing effective care pathways, in addition to the argument that current cognitive screening tools^2^ (and practices) may be unsuitable for identifying possible cases of dementia in custodial settings (Brooke et al., 2020), our research group has undertaken a multi-phased study in a Canadia facility to assess the performance of several cognitive screening tools for detecting suspected cases of dementia when compared with a formal diagnostic assessment (i.e., diagnosis by a geriatrician). To that end, the purpose of this systematic review was to synthesize findings from studies that have examined cognitive impairment and dementia among older people in custody using cognitive screening tools to provide a comprehensive overview of the evidence gathered thus far. While there are recent reviews on cognitive impairment and dementia in prison (Brooke et al., 2020; du Toit et al., 2019; Peacock et al., 2019; Treacy et al., 2024), to our knowledge, no reviews have specifically assessed the evidence on cognitive screening tools.^3^

## Methods

### Search Strategy and Data Extraction

For this review, focus was exclusively on studies that involved the use of cognitive screening tools within custodial settings. Systematic literature review procedures followed the Preferred Reporting Items for Systematic Reviews and Meta-Analyses (PRISMA) guidelines (Liberati et al., 2009; Page et al., 2021). The first step involved conducting Boolean searches (completed in May 2023) using a combination of keywords and phrases to scan electronic databases for studies that applied cognitive screening tools and practices to assess dementia and cognitive impairment among older people in custody. Information sources included electronic databases that index journals in the cognitive, health, and applied sciences (i.e., PsycINFO, MedLine, PubMed, and Academic Search Complete). The yield of records from electronic database searches were further supplemented through the identification of studies via other methods (e.g., handsearching reference lists) to ensure any eligible studies were not excluded. Table 1 summarizes the search terms, databases, and corresponding number of records identified. It should be noted that the Sociological Abstracts database was also searched (producing a total of 2,126 records); however, this database and corresponding records were excluded as most search results were irrelevant to the study topic and the records that were relevant were duplicates.

**Table 1.** Search Terms, Databases, and Number of Identified Records

Search terms	Databases and records			
	PsycINFO	MEDLINE	PubMed	Academic search complete
Aging offender OR older inmate OR criminals OR incarcerated individual OR prisoner OR people in custody OR persons in custody AND dementia OR Lewy body OR Alzheimer OR vascular dementia OR frontotemporal OR memory loss OR cognitive decline AND screening OR assessment	146	16	12	52
Offender OR inmate OR criminals OR incarcerated OR prisoner OR people in custody OR persons in custody AND dementia OR Lewy body OR Alzheimer OR vascular dementia OR frontotemporal OR memory loss OR cognitive decline AND screening OR assessment	146	19	14	60
Prison OR custodial OR forensic OR custodial health OR correctional facility AND dementia OR Lewy body OR Alzheimer OR vascular dementia OR frontotemporal OR memory loss OR cognitive decline AND screening OR assessment	170	74	24	167
Indigenous OR Inuit OR Métis OR first nation OR aboriginal OR native AND offender OR inmate OR prisoner OR incarcerated individual OR people in custody OR persons in custody AND dementia OR Lewy body OR Alzheimer OR vascular dementia OR frontotemporal OR memory loss OR cognitive decline AND screening OR assessment	0	0	1	3

All records identified through the electronic database searches were uploaded into Covidence (https://www.covidence.org/) for the removal of duplicates, title and abstract screening, and full-text review. For title and abstract screening, as well as full-text review, records were compared with inclusion and exclusion criteria to narrow results to publications that relate specifically to the objective of this study. Inclusion criteria included peer-reviewed studies with primary data assessing dementia and cognitive impairment among people in custody using screening tools; focused on older custodial populations (i.e., 45 years of age and older); were conducted in high-, middle-, and low-income countries; specifically examined dementias or age-related cognitive impairment; and, were written in English and published between 2000 and 2025. Exclusion criteria included non-peer-reviewed studies; studies focused on younger people in custody (i.e., younger than 45 years of age); studies that did not directly assess dementia or cognitive impairment using screening tools; publications on psychiatric illnesses without specifically identifying dementia or cognitive impairment; studies that examined community-based correctional populations or free citizens; articles without primary data, theses/dissertations, books, technical reports, literature reviews, news articles, opinion and discursive pieces; and, research papers on neurocognitive issues unrelated to the focus of this study or examining other primary conditions presenting with cognitive impairment (e.g., intellectual disabilities, learning disorders, motor disorders, stroke, traumatic brain injury, Fetal Alcohol Spectrum Disorder, severe depression, etc.).

The initial search (and removal of duplicates), screening of titles and abstracts, full-text review, and extraction of data was conducted by the first and second authors (MI and BS). Co-authors were consulted following title and abstract screening, as well as full-text review, and full consensus was required for including or excluding studies at each stage. There were no disagreements. Final articles were selected following full-text review according to the above criteria and key objectives of the current review, and information from each eligible study was extracted and synthesized in Table 2 by the first and second authors (MI and BS). This included providing publication details and summarizing information pertaining to study aims, settings, participants and methods, assessment tools, key findings, and conclusions. Co-authors verified all information extracted from the eligible studies for completeness and accuracy.

**Table 2.** Investigations of Cognitive Assessment and Dementia Screening Tools Among Older People in Custody

Study	Keywords	Synopsis
Perez et al. (2021)	Cognitive impairment, race differences, aging, incarceration	Country: United States
		Aim: To assess cognitive impairment (global cognition and executive functioning) in older men in custody, according to education and race
		Setting: State prison
		Participants and methods: 239 older men in custody (aged 50 years and older) with varying racial/ethnic backgrounds were evaluated for cognitive impairment. Of these participants, 38% were white, 41% were black, and 21% were Hispanic/other. In-person interviews were conducted to administer the assessment tool
		Assessment tool: Montreal cognitive assessment (MoCA)
		Results: Overall, 62.8% of participants met the criteria for cognitive impairment when standard scoring and cut-off points were used, whereas 38.5% of participants met the criteria when scoring and cut-off points were adjusted based on education and race/ethnicity. This shift was mostly due to the significant reduction in the percentage of black individuals who met the criteria for cognitive impairment after applying education- and race-specific cut points (62.6% vs. 19.2%). After adjusting for the key demographic factors, fewer white individuals met the criteria for cognitive impairment (51.1% vs. 36.7%), while the percentage of Hispanics/Others remained unchanged (84% before vs. 80% after)
		Conclusion: Acknowledging key demographic characteristics (e.g., education and race/ethnicity) when using the MoCA resulted in notable differences in the number of older people in custody who met the criteria for cognitive impairment. This highlights the need for a validated assessment tool for this diverse population
Stoliker et al. (2022)	Dementia, older adults, forensic psychiatric hospital, community reintegration, health care, social care	Country: Canada
		Aim: The primary focus was to determine the prevalence of dementia (i.e., probable cases) among older individuals in custody in a forensic psychiatric facility. A secondary focus was to identify correctional health care professionals’ perspectives about the older individuals on their caseload and training/education to support older people in custody
		Setting: A forensic psychiatric hospital that provides psychiatric assessment, rehabilitation, and treatment to federally sentenced individuals
		Participants and methods: In total, 29 older people in custody (aged 46 to 80 years; *M* = 59, *SD* = 8.67) were screened for dementia (3.4% identified as female and 55% reported indigenous status), and 20 correctional health care professionals (i.e., 8 social workers and 12 nurses) completed a self-report survey
		Assessment tool: Modified version of the community screening instrument for dementia (CSI'D')
		Results: Overall, 45% of older individuals screened positive for dementia using the modified version of the community screening instrument for dementia (CSI'D'). In addition, 35% of social workers and 25% of nurses suspected dementia in at least one older person on their caseload, with findings further highlighting adequate levels of agreement between health staffs’ perception of the presence or absence of dementia and the CSI‘D’ assessment. Most staff reported they did not have specialized training on dementia (95%) or other specialized training about older people in custody (100%), nor did they feel they had adequate training/education to support older individuals on their caseload (60%)
		Conclusion: Enhanced cognitive screening tools and practices, adequate staff training, and a collaborative treatment approach are essential for managing older individuals in custody at risk of neurocognitive issues such as dementia
Forsyth et al. (2020)	Dementia, cognitive impairment, older prisoners, prison	Country: England and wales
		Aim: The focus of this study was to: (a) Determine the prevalence of dementia and mild cognitive impairment among older people in custody in England and wales, as well as the needs of these individuals for health and social care; and, (b) validate the six-item cognitive impairment test (6-CIT) for routine use in prisons for early detection of dementia or mild cognitive impairment
		Setting: All women’s prisons (*n* = 10) and a representative sample of men’s prisons (*n* = 11) across England and wales
		Participants and methods: 869 older people in custody aged 50 years and older (596 men and 273 women) randomly drawn from the selected prisons. In-person interviews were conducted to first screen participants using the MoCA and, subsequently, those testing positive on the MoCA (i.e., a score of ≤23) were interviewed using the ACE-III. A proportion of the sample also completed the 6-CIT for validation purposes. In addition, over 100 multidisciplinary staff members involved in prison management, health services, and social care services completed questionnaires about service delivery and staff training
		Assessment tool: Montreal cognitive assessment (MoCA), addenbrookes cognitive examination – third revision (ACE-III), and the six-item cognitive impairment test (6-CIT)
		Results: Overall, the prevalence of suspected dementia and mild cognitive impairment (combined) in the prison population of England and wales was estimated to be 8%. Among older individuals in custody, the MoCA performed better than the 6-CIT. In addition, prison staff had limited training in dementia and mild cognitive impairment, which negatively impacted their interaction with older individuals in custody living with these neurocognitive issues
		Conclusion: Adequate staff training, improved social and health services, and additional research on the validation of cognitive screening tools will help improve the provision of care to older individuals in custody living with mild cognitive impairment and dementia
Mantell et al. (2023)	Serious game, gamification, cognitive assessment, prison, older adults, older prisoners, game design, self-determination theory	Country: Australia
		Aim: To examine user design preferences and acceptability of a game-based cognitive assessment tool for older people in custody from the perspective of these individuals
		Setting: Prisons (of unknown type)
		Participants and methods: A qualitative study involving 20 older people in custody (aged 50+ for non-indigenous peoples and 45+ for indigenous peoples) from three prisons in New South Wales, Australia. Focus groups were conducted via virtual sessions
		Assessment tool: *Development of an innovative game-based cognitive assessment tool*
		Results: Qualitative data were analyzed and interpreted according to the self-determination theory (SDT), and three themes emerged. The “goldilocks” theme emphasized participants’ collective desire for an individualized optimal level of difficulty in serious gameplay. The “childish graphics” theme raised the importance of avoiding immature and childlike gameplay features, as some older users felt that these could be condescending. The “balanced diet” theme highlighted strong user preference for meaningful choice and variety in any serious game-based cognitive assessment to maximize in-game autonomy
		Conclusion: Findings from this study provide a fresh view into designing unique and compelling cognitive assessment tools and practices for older people in custody. Further research on the efficacy of a game-based cognitive assessment tool for prison settings would be welcomed
Verhülsdonk et al. (2023)	Cognition, elderly prisoners, forensic psychiatric health care, correctional health care, mental health, dementia	Country: Germany
		Aim: To compare the prevalence of cognitive impairment between the prison and forensic hospital populations in north Rhine-Westphalia, Germany
		Setting: Prisons (of unknown type) and forensic hospitals
		Participants and methods: The final sample included 57 older people in custody (men) from five different prisons and 34 forensic inpatients (men) from five different forensic hospitals in north Rhine-Westphalia, Germany (all participants were aged 60 years and older). In-person interviews were conducted to administer two cognitive screening tools to both sample groups. Women were not included in the study as these establishments were note accessible to the researchers
		Assessment tool: Mini-mental state examination (MMSE) and DemTect
		Results: When using the MMSE, 36.8% of people in custody and 17.6% of inpatients showed signs of mild cognitive impairment, 23.5% of inpatients had mild symptoms of dementia, 5.3% of people in custody and 20.6% of forensic inpatients were classified as having moderate symptoms of dementia, and only one participant in the forensic inpatient sample showed signs of severe dementia. In contrast, the DemTect found that 15.8% of people in custody and 20.6% of forensic inpatients showed dementia-like symptoms, with 19.3% of people in custody and 32.4% of forensic inpatients with mild cognitive impairment
		Conclusion: Further research should explore the development of a more specialized cognitive assessment tool validated for use in correctional (and forensic) facilities
Combalbert et al. (2018)	Cognitive impairment, aging, prison, older prisoners, perceived health	Country: France
		Aim: To examine the cognitive performance of older people in custody (men) and its effect on their perceived health and quality of life
		Setting: Prisons (of unknown type)
		Participants and methods: The final sample included 138 men aged 50 years and older from seven French prisons, as well as 138 men of a similar age drawn from the general population. In-person interviews were conducted to administer the cognitive screening tool. Participants also received self-report questionnaires to assess overall health (Nottingham health profile) and subjective state of mental health and quality of life (the brief world health organization quality of life questionnaire)
		Assessment tool: Mini-mental state examination (MMSE)
		Results: In terms of cognitive function based on the MMSE, 26 (18.8%) older people in custody obtained a score equal to or less than 23, which is suggestive of cognitive impairment. However, only one (0.72%) individual in the comparison group obtained a similar score. Furthermore, no statistically significant associations were found in either group with respect to the relationship between cognitive decline and life satisfaction or perceived health
		Conclusion: It is crucial that older people in custody receive a thorough evaluation for cognitive impairment
Verhülsdonk et al. (2021)	Mental health, dementia, correctional health care, elderly prisoners, cognitive dysfunction, mild cognitive impairment	Country: Germany
		Aim: To gather the first empirical information on the cognitive condition of older people in custody in Germany and to investigate relationships between cognitive performance and sociodemographic, clinical, and imprisonment factors
		Setting: Prisons (of unknown type)
		Participants and methods: The final sample included 58 older people in custody (aged 60 and older) from five different prisons in north Rhine-Westphalia, Germany. In-person interviews were conducted to assess global cognition through the administration of two cognitive screening tools
		Assessment tool: Mini-mental state examination (MMSE) and DemTect
		Results: When using the MMSE with age- and education-corrected z-scores, 36.9% of participants had scores that indicated marginal or impaired global cognition. When using the DemTect, 41.4% of participants showed signs of cognitive impairment. Correlation analyses revealed statistically significant associations between cognitive scores and age, education, sentence duration, and duration of current incarceration
		Conclusion: There is an urgent need for effective management of older people in custody with cognitive impairment, including routine cognitive testing and treatment of symptoms according to clinical recommendations and best practices
Ahalt et al. (2018)	Cognitive impairment, cognition, jail, criminal justice, vulnerable population	Country: United States
		Aim: To ascertain the prevalence of cognitive impairment among older people in custody
		Setting: Urban county jail
		Participants and methods: The final sample included 310 older people in custody (aged 55 and older) in one urban county jail. In-person interviews were conducted to administer the cognitive screening tool
		Assessment tool: Montreal cognitive assessment (MoCA)
		Results: Using the MoCA, it was found that 70% of participants showed signs of cognitive impairment. These individuals (i.e., those with signs of cognitive impairment) were more likely to be non-white (81%) and reported having fair to poor health (54%). Over the course of six months, multiple emergency room visits (32%), hospitalizations (35%), and recurrent arrests (45%) were all related to poor cognition
		Conclusion: Older people in custody frequently show signs of cognitive impairment, which may be linked to poor health and criminal justice outcomes. To identify and treat cognitive impairment, a strategy should be implemented that focuses on specialized geriatric care within the jail setting and beyond. More research is required to accurately identify cognitive impairment and its effects in this population
Forsyth et al. (2025)	Dementia, prevalence, prisons	Country: England and wales
		Aim: A sub-analysis of Forsyth et al. (2020), the focus of this study was to: (a) Determine the prevalence of dementia and mild cognitive impairment among older people in custody in England and wales; and, (b) identify risk of harm to self and others, as well as activity of daily living needs and social networks, of people in custody with suspected mild cognitive impairment and dementia
		Setting: All women’s prisons (*n* = 10) and a representative sample of men’s prisons (*n* = 11) across England and wales
		Participants and methods: 869 older people in custody aged 50 years and older (596 men and 273 women) randomly drawn from the selected prisons. In-person interviews were conducted to first screen participants using the MoCA and, subsequently, those testing positive on the MoCA (i.e., a score of ≤23) were interviewed using the ACE-III. In addition, several standardized instruments were administered to those testing positive on the MoCA to assess risk of harm to self and others, activities of daily living needs, mental health needs, history and symptoms of brain injury, and social networks
		Assessment tool: Montreal cognitive assessment (MoCA) and addenbrookes cognitive examination – Third revision (ACE-III)
		Results: One hundred (12% of total sample) participants screened positive on the MoCA and 70 (8% of total sample) went on to screen positive on the ACE-III for mild cognitive impairment or dementia. Across the entire sample, the prevalence of dementia was estimated to be 7% (95% CI [5.5, 8.9]), with those aged 70 years and older exhibiting the highest rates at 11.0% (95% CI [7.4, 16.2]), followed by those aged 50–59 (6.1%; 95% CI [4.2, 8.8]) and those aged 60–69 (5.0%; 95% CI [2.9, 8.5]). When disaggregated according to gender, the prevalence of dementia was estimated to be 7.0% for men (95% CI [5.2, 9.4]) and 6.0% for women (95% CI [3.8, 9.5]). The prevalence of mild cognitive impairment was estimated to be 0.8% (95% CI [0.4, 1.7]). Among those who tested positively on the MoCA, 46% (*n* = 32) had a high or very high risk of harm to self and others, and only 3% (*n* = 2) had a diagnosis of dementia in their prison healthcare records. Generally, those who screened positively on the ACE-III exhibited a high level of physical and mental health needs, as well as low level of social support. Compared with people of the same age living in the community, the prevalence of dementia among people in custody was 3-4 times higher for those aged 60–69 and 3.5 times higher for those aged 70 and older
		Conclusion: The provision of care and specialized services for older people in custody living with possible dementia and mild cognitive impairment requires further attention, and cognitive screening tools will need validation for use in prison populations (including exploring adjustments to scoring procedures)

Furthermore, eligible studies were examined under three major themes: (1) use of screening tools with modification for the correctional setting; (2) use of screening tools without modification for the correctional setting; and, (3) innovative advances in cognitive screening tools and practices for correctional settings. The first two themes were developed by the authors *a posteriori* and aimed to establish how cognitive screening tools have been used within custodial settings given that these instruments have primarily been developed for, and used with, community-based populations. The third theme was developed *ad hoc* following a review of the literature. Following data extraction and the development of study synopses (Table 2), we categorized and narratively reviewed the articles according to the abovementioned themes. All authors discussed and agreed upon the three major themes that guided the categorization and narrative review of eligible studies. It is important to note that we did not use a specific system or framework to create the themes or to guide the narrative review; rather, studies were critically assessed according to their relevance to one or more of the major themes.

## Results

### Identified Studies

**Figure 1.**
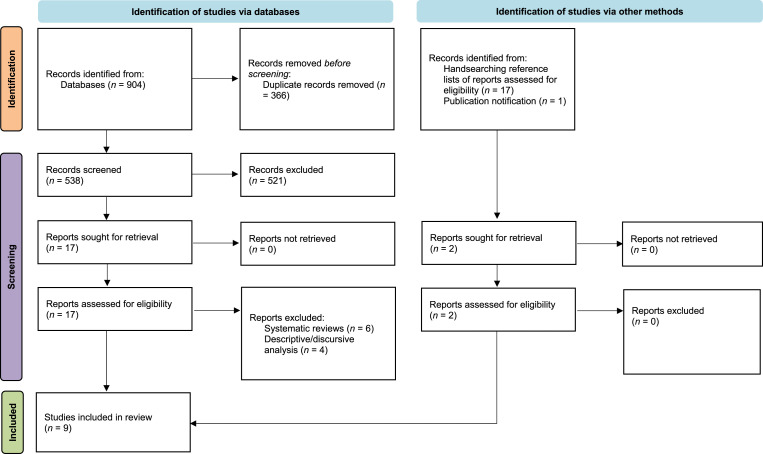
PRISMA Summary (informed by Page et al., 2021)

Figure 1 presents the PRISMA summary, highlighting the selection of eligible studies for the current systematic literature review. With respect to electronic database searches, a total of 904 records were identified, from which 366 duplicates were removed. This left 538 records for title and abstract screening. Five hundred and twenty-one records were removed through title and abstract screening, leaving 17 records for full-text review. Ten records were excluded following full-text review as they did not meet eligibility criteria (six were systematic reviews and four were descriptive or discursive analyses). A total of 7 studies identified through database searches met the eligibility criteria following full-text review. With respect to the identification of studies via other methods, one article was included after handsearching the reference lists of the 17 studies included for full-text review. One additional article was included following the publication of a study at the time this paper was being revised (early 2025), which had relevance to the current systematic review. Overall, the limited number of records highlights the paucity of resources on this topic. Table 2 presents synopses for each of the 9 studies that were eligible for inclusion.

## Discussion

This section critically examines the studies identified by the systematic review according to their direct investigation of cognitive impairment and dementia among older people in custody using cognitive screening tools and practices. Discussion is structured according to the three major themes outlined in the Methods section.

### Screening Tools Modified for Correctional Settings

Given the argument that current cognitive screening tools are likely inadequate when it comes to detecting probable cases of dementia among older people in custody (Brooke et al., 2020), several studies have modified existing tools to suit the correctional context (e.g., by modifying scoring procedures or specific items). The Montreal Cognitive Assessment (MoCA) was modified and used in four studies (Ahalt et al., 2018; Forsyth et al., 2020, 2025; Perez et al., 2021), and the Community Screening Instrument for Dementia (CSI'D') was modified and used in one study (Stoliker et al., 2022).

#### Montreal Cognitive Assessment (MoCA)

The Montreal Cognitive Assessment (MoCA) was developed in 2005 (Davis et al., 2021) and is a 30-item measure that takes 15 min to administer and examines executive functioning, verbal memory, attention, orientation, language, and other areas (Perez et al., 2021). According to Forsyth et al. (2020, 2025), a score greater than or equal to 26 points is considered “normal” cognitive function for community-based populations. However, several studies have questioned this cut-off score for use with disadvantaged populations (Forsyth et al., 2020, 2025). The MoCA is frequently used in clinical settings (Nasreddine et al., 2005; Paraizo et al., 2016; Smith et al., 2007; Tiffin-Richards et al., 2014) and has a high sensitivity for detecting mild cognitive impairment (MCI) and dementia in various racial groups, as well as vulnerable populations, despite not having been previously validated for use in prison populations (Milani et al., 2018).

In the Perez et al. (2021), Forsyth et al. (2020, 2025), and Ahalt et al. (2018) studies, adjustments were made to the scoring procedure for the MoCA. Although those scoring below 26 were deemed to have cognitive impairment in the Perez et al. (2021) study, participants’ MoCA scores were adjusted upward by one point if they had 12 years of education. Findings from this study suggested that racial/ethnic differences still exist despite using scoring adjustments to determine cognitive impairment, raising questions about the validity of ignoring key demographic factors (e.g., education and race/ethnicity) when measuring cognitive impairment in a carceral context. In the Ahalt et al. (2018) study, a positive screen was considered to be a score of less than 25 as a meta-analysis revealed that this lower cut-off is optimal for detecting mild cognitive impairment (Ciesielska et al., 2016), and others have suggested that a cut-off score of 26 exaggerates impairment in certain populations (Rossetti et al., 2011; Sink et al., 2015). In this study (Ahalt et al., 2018), scores were adjusted upward (by one point) for individuals who had not completed high school level education. Notably, for both the Perez et al. (2021) and Ahalt et al. (2018) studies, the predictive accuracy (i.e., sensitivity and specificity) of the modified versions of the MoCA was not mentioned. In the Forsyth et al. (2020, 2025) studies, a cut-off score of 23 points was utilized as it was determined that a high percentage of false positives could have resulted from using a cut-off score of 26 points. Forsyth et al. (2020) noted that the modified version of the MoCA had a sensitivity of 89% and a specificity of 84% when screening for dementia and MCI.

#### Community Screening Instrument for Dementia (CSI’D’)

The Community Screening Instrument for Dementia (CSI’D’) (Hall et al., 1993, 1996, 2000; Hendrie et al., 1995; Unverzagt et al., 1999) is a two-part measure that includes a participant interview and informant survey. To assess people in a custodial context more accurately, Stoliker et al. (2022) modified items on the original CSI’D’ (in cooperation with the tool’s original developers) to flag older Indigenous and non-Indigenous persons in custody at risk for dementia and who require further clinical assessment. The modified version of the participant interview was a 46-item measure (with 33 scored items) that takes approximately 25 minutes to administer and examines memory (recall), language expression, registration, attention and calculation, orientation to place and time, language comprehension, and praxis. The modified version of the informant survey was a 31-item measure (with 30 scored items) that takes approximately 15 min to complete and asks primary caregivers (e.g., nurses) about the participant according to memory and cognition, activities of daily living, and miscellaneous problems. Accordingly, the CSI‘D’ contains a Cognitive Score obtained from the participant interview and Informant Score obtained from the informant survey (Hall et al., 1993, 1996, 2000). A Discriminant Score, which represents the likelihood of possible dementia, is calculated according to the Cognitive Score and Informant Score (Hall et al., 1996, 2000). CSI’D’ scores can also be calculated without the Informant Score, however, there is better reliability with it. As a culturally sensitive tool, the CSI'D’ has scoring guidelines for both Indigenous and non-Indigenous peoples (Hall et al., 2000; see also Stoliker et al., 2022). Kerodal et al. (2020) thoroughly describe the elements of the modified CSI’D’, as well as scoring procedures.

With respect to its development, the CSI’D’ was created in consultation with Cree Elders in Manitoba, Canada and was then validated with a sample of Manitoban Cree People and non-Indigenous people in Winnipeg, Canada (Hall et al., 1993; Hendrie et al., 1995). Since then, the CSI’D’ has been examined in validation studies using a wide range of community-based samples, including racialized groups in the United States, Asia, South America, the Caribbean, the Middle East, and Africa (Davoudkhani et al., 2019; Hall et al., 1993, 1996; Hendrie et al., 1995; Unverzagt et al., 1999). However, the CSI’D’ had not been used with people in custody prior to the study by Stoliker et al. (2022). The modified version of the CSI’D’ has also yet to be validated for correctional populations and, therefore, the accuracy of estimates identified in Stoliker et al. (2022) is unknown.

### Screening Tools Without Modification for Correctional Settings

Several studies utilized cognitive screening tools without modification for the correctional setting, which included the Mini-Mental State Examination (MMSE) (Combalbert et al., 2018; Verhülsdonk et al., 2021, 2023), Addenbrookes Cognitive Examination – Third Revision (ACE-III) (Forsyth et al., 2020, 2025), DemTect (Verhülsdonk et al., 2021, 2023), and Six-Item Cognitive Impairment Test (6-CIT) (Forsyth et al., 2020).

#### Mini-Mental State Examination (MMSE)

The Mini-Mental State Examination (MMSE) assesses short-term verbal memory, immediate and delayed recall, orientation to time and place, attention, visual construction, math, and language (Folstein et al., 1975) and takes approximately 10 to 15 min to administer. A participant may receive up to 30 points, with higher scores suggesting better cognitive function (Verhülsdonk et al., 2023). The cut-off for cognitive impairment is set at 27 points, with mild symptoms of dementia indicated by 20 to 26 points, moderate symptoms of dementia by 10 to 19 points, and severe dementia by 10 points or lower (Deuschl & Maier, 2016). Previous studies have raised concerns about the validity of traditional screening tools like the MMSE in the context of prisons, particularly regarding questions about orientation to time and place (Grassian, 2006). Notably, the MMSE has not been previously validated for use in prison populations. The MMSE also exhibits poor sensitivity in detecting early dementia, does not examine executive function, and scores may be affected by age, race, and education (Velayudhan et al., 2014). The studies by Verhülsdonk et al. (2021, 2023) and Combalbert et al. (2018) used the MMSE and, in all three studies, sensitivity and specificity were not described. Although, due to the limitations of the MMSE, the authors suggested that a more specialized tool is required for the custodial context.

#### Addenbrookes Cognitive Examination – Third Revision (ACE-III)

According to Hsieh et al. (2013), the ACE-III is one of the most frequently used cognitive screening tools and is validated for the assessment of dementia and other neurological illnesses. However, the ACE-III has not yet been validated for use in non-clinical settings, such as prisons (Forsyth et al., 2020, 2025; Velayudhan et al., 2014). This tool takes approximately 15 min to administer and includes tasks that measure verbal fluency, memory, language, and visuospatial ability, with a maximum achievable score of 100 (Hsieh et al., 2013). The studies by Forsyth et al. (2020, 2025) used the ACE-III to identify the prevalence of MCI and dementia among older people in custody in England and Wales. In this research, the cut-off values of 82 and 88, respectively, suggested probable cases of dementia and MCI. Research has revealed that the cut-off scores of 82 for dementia and 88 for MCI have 93–100% sensitivity and 96–100% specificity rates (Hsieh et al., 2013; Noone, 2015). Findings from Forsyth et al. (2020, 2025) indicated that 8% of participants screened positive for mild cognitive impairment or dementia using the ACE-III. Specifically, the prevalence of dementia was estimated to be 7%, whereas the prevalence of mild cognitive impairment was estimated to be 0.8%. Alternatively, 12% screened positive on the (modified) MoCA, potentially suggesting more conservative scores for the ACE-III (Forsyth et al., 2020, 2025).

#### DemTect

The DemTect is a dementia screening tool (Kalbe et al., 2004) that takes approximately 10 min to administer (Velayudhan et al., 2014). It is comprised of five smaller tasks that assess working memory, executive function, short- and long-term verbal memory, semantic fluency, and numerical conversion. With respect to scoring, age-appropriate cognitive function is between 13 and 18 points, mild cognitive impairment is between 9 and 12, and symptoms of dementia is 8 points or lower. The DemTect has a better sensitivity than the MMSE for the early detection of cognitive decline (Beyermann et al., 2013; Kalbe et al., 2013; Scheurich et al., 2005). However, it has a relatively low specificity (72%) and has only been validated for use in clinical settings (Larner, 2007), with no studies on its validity in prison populations. It was used in the Verhülsdonk et al. (2021, 2023) studies and, in both cases, there was a high rate of false positives. Again, the authors advised using a more specialized tool for the custodial context.

#### The Six-Item Cognitive Impairment Test (6-CIT)

The 6-CIT (Brooke & Bullock, 1999) is a brief dementia screening test, which takes approximately 3 to 5 min to administer and includes questions pertaining to the day, time, and year as well as assesses the individual’s memory of a five-part address, ability to list numbers, and capacity to list the months of the year in reverse (Forsyth et al., 2020). In the Forsyth et al. (2020) study, the 6-CIT was used to compare its clinical efficacy with that of the MoCA in detecting individuals with possible dementia and MCI. The study also attempted to validate the 6-CIT so that it could be used to screen for dementia and MCI in custodial settings. Unfortunately, its low performance compared with the MoCA (i.e., 6-CIT screened 8.3% less individuals with dementia and MCI than the MoCA) led to the conclusion that the MoCA was a better screening tool and that the 6-CIT was unsuitable for use in a correctional context. However, further detail on the sensitivity and specificity of the 6-CIT was not disclosed.

### Innovative Advances in Cognitive Screening Tools and Practices for Correctional Settings

One of the most interesting solutions proposed to enhance the user experience of formerly tedious tasks is gamification (Wiley et al., 2021). To boost interest and motivation, the gamification process involves integrating game design features like challenges, scoring, aesthetics, and storylines into non-game situations such as psychometric testing. Serious games are often referred to as gamified tasks that are integrated into an immersive user experience (Mantell et al., 2023). Users of serious games are given distinctive and engaging environments to accomplish tasks that go beyond simple amusement (Lumsden et al., 2016). The examination of serious games for cognitive assessment is a developing field of study. Although utilizing conventional cognitive evaluation instruments has been shown to have its advantages, such as high sensitivity to neurological disorders and demonstrated psychometric validity (Dementia Australia, 2022; Nasreddine et al., 2005; Salmon & Bondi, 2009), users frequently view them as monotonous and repetitive (DeRight et al., 2015). Importantly, the reliability of performance data can be impacted by this perception (Khaleghi et al., 2021; Wiley et al., 2021) and can lower work engagement (Friehs et al., 2020).

If gamification claims to raise user incentive to complete a cognitive assessment but does not deliver on this claim, it is vital to figure out what influences an individual’s motivation. Self-determination theory (SDT) is a well-known theory that is used to identify an individual’s level of motivation (Mantell et al., 2023). According to SDT, the fundamental needs of competence (i.e., experiencing mastery over challenges), autonomy (i.e., acting on one’s own volition), and relatedness (i.e., engaging in meaningful social relationships) must be met in order to generate motivation, enjoyment, and overall well-being (Mantell et al., 2023). SDT has been implemented to understand the motivation to play digital games (Denson et al., 2022; Przybylski et al., 2010; Ryan et al., 2006), in addition to aiding researchers in understanding motivation generally. According to Uysal and Yildirim (2016), the theory has helped to explain how game elements link to fundamental psychological demands and how game design and development choices might consequently influence player motivation and enjoyment. Ryan et al. (2006) further demonstrated that specific features and gameplay in games directly increase motivation.

Mantell et al. (2023) aimed to improve insight into user design preferences and acceptability of a game-based cognitive assessment tool for older people in custody within the context of both the prison environment and future transition back to the community (which is currently undergoing more research). In Mantell et al.’s (2023) study, older people in custody were asked about their design preferences for a serious game with a qualitatively based cognitive assessment. From the focus groups, three themes emerged. First, the “Goldilocks” theme suggested that players shared desire for an individually optimal amount of challenge in serious gameplay. Second, the “avoiding childish graphics” theme highlighted the significance of avoiding immature and childlike gameplay features, as they can feel condescending to some users. Third, and finally, the “balanced diet” theme emphasized the strong user preference for meaningful choice and variety in any serious game-based cognitive assessment. These themes offer fresh perspectives into the preferences of older people in custody for the design of (game-based) cognitive assessments, whose opinions have thus far largely gone unheard in the development of these tools. Taken together, by situating these themes in the SDT framework and game design research, developers for cognitive assessment tools and practices will have a solid foundation from which to create cognitive assessments that are motivating, culturally appropriate, enjoyable, and compelling for older individuals within the unique context of prison environments.

## Conclusion

The number of older and aging individuals in custody is rising worldwide and, with that, correctional systems are faced with the challenge of addressing the complex health and social needs of this growing demographic, including neurocognitive issues such as cognitive impairment and dementia. This systematic review synthesized evidence from 9 studies across several high-income countries that have applied cognitive screening tools and practices to assess cognitive impairment and dementia among older people in custody. Several cognitive screening tools and practices have been used, including those modified for correctional settings (i.e., modified MoCA and CSI’D’), those used without modification (i.e., MMSE, ACE-III, DemTect, and 6-CIT), and innovative methods for cognitive assessment in correctional settings (Mantell et al., 2023). However, previous research has primarily used cognitive screening tools to estimate prevalence rates and, apart from one known study that aimed to validate an instrument to screen for dementia and cognitive impairment in custodial populations (Forsyth et al., 2020), cognitive screening tools and practices have yet to be fully validated for custodial settings. Based on the current review, we offer some recommendations to advance research and practice regarding cognitive assessment within the carceral context.

(1) Correctional systems should implement cognitive screening as part of standard correctional healthcare policy and practice (Brooke et al., 2020; Peacock et al., 2019).
  • Early detection of possible cases of cognitive impairment or dementia will be essential to effectively manage older people in custody living with these neurocognitive issues, including the implementation of care pathways that provide individualized and specialized supports and services (Forsyth et al., 2020, 2025; MacRae et al., 2024; Treacy et al., 2024). Cognitive screening should be routinized and, when positive, followed by a thorough evaluation by a physician to establish diagnosis and treatment. Correctional and healthcare staff will also require specialized training to support older people in custody, including training on the administration and interpretation of cognitive assessments (Forsyth et al., 2025; Stoliker et al., 2022).
(2) While cognitive screening tools continue to be used in correctional research and practice, the reliability and validity of these instruments have not been rigorously examined. To effectively identify probable cases of cognitive impairment and dementia and subsequently provide specialized care, researchers must continue to prioritize the development and validation of cognitive screening tools and practices tailored to the correctional setting.
  • Future investigations are required to assess the reliability and validity of cognitive screening tools for custodial populations, which should consider: (1) whether existing instruments should be modified (e.g., adjustments to scoring procedures, revising items, etc.), if existing instruments are suitable as is, or if specialized cognitive assessment tools and practices are required for the custodial setting; (2) what instrument(s) perform best in predicting possible cases of dementia or cognitive impairment (i.e., when compared against a formal diagnostic assessment); (3) whether a combination of instruments should be used to account for the varying strengths and limitations of screening tools; and, (4) assessing instruments for capability of early detection.

## ORCID iD

Bryce E. Stoliker https://orcid.org/0000-0002-3978-9083

## Notes

1. Older and aging people in custody are generally defined as those aged 50 years and over for non-Indigenous populations and those aged 45 years and over for Indigenous Peoples (Baidawi et al., 2011; Merkt et al., 2020).

2. Cognitive screening tools refer to rapid instruments that aim to uncover potential neuropsychological dysfunction, mild cognitive impairment, or dementia and may be used by healthcare professionals to decide whether additional diagnostic and therapeutic procedures are necessary (Kalbe et al., 2013).

3. Some reviews include discussion of cognitive screening tools (e.g., see Brooke et al., 2020; du Toit et al., 2019), but only as a component of a broader review on the issue of dementia in prison.
